# Rapid Decrease in HDL-C in the Puberty Period of Boys Associated with an Elevation of Blood Pressure and Dyslipidemia in Korean Teenagers: An Explanation of Why and When Men Have Lower HDL-C Levels Than Women

**DOI:** 10.3390/medsci9020035

**Published:** 2021-05-24

**Authors:** Kyung-Hyun Cho, Jae-Ryong Kim

**Affiliations:** 1 Korea Research Institute of Lipoproteins, Medical Innovation Complex, Daegu 41061, Korea; 2 LipoLab, Yeungnam University, Gyeongsan 38541, Korea; 3 Department of Biochemistry and Molecular Biology, Smart-Aging Convergence Research Center, College of Medicine, Yeungnam University, Daegu 42415, Korea; kimjr@med.yu.ac.kr

**Keywords:** high-density lipoproteins-cholesterol, puberty, boys, life-expectancy, blood pressure, dyslipidemia

## Abstract

Low serum high-density lipoproteins-cholesterol (HDL-C) levels and high blood pressure are linked to each other and are recognized as independent risk factors of cardiovascular disease and dementia. HDL can cross the blood–brain barrier to remove amyloid plaque and the blood–testis barrier to supply cholesterol for spermatogenesis, but LDL cannot. During the teenage period, between 10 and 19 years of age, the systolic blood pressure (BP) increased gradually to 7.9% in boys (*p* < 0.001), but not in girls (*p* = 0.141). The boys’ group showed a remarkable decrease in the total cholesterol (TC) and HDL-C from 10 to 15 years of age (*p* < 0.001). After then, the TC level increased again at 19 years of age to the previous level (*p* < 0.001). On the other hand, the HDL-C level at 19 years of age in the boys’ group was not restored to the previous level at 10 years of age. The girls’ group maintained similar TC (*p* < 0.001) and HDL-C (*p* < 0.001) levels from 10 to 19 years of age. These results suggest there was a remarkable difference in cholesterol consumption, particularly in the HDL-C level between boys and girls during the pubertal period. Correlation analysis showed an inverse association between the HDL-C level and SBP in boys (r *=* −0.133, *p* < 0.001) and girls (r *=* −0.065, *p* = 0.009) from 10 to 19 years of age. Interestingly, only the boys’ group showed an inverse association with the diastolic BP (r *=* −0.122, *p* < 0.001); the girls’ group did not have such an association (r *=* −0.016, *p* = 0.516). In conclusion, the boys’ group showed a sharp decrease in the HDL-C level from 10 to 15 years of age, whereas the girls’ group showed an increase in the HDL-C level during the same period. These results explain why men have a lower serum HDL-C level than women in adulthood.

## 1. Introduction

A low serum HDL-C level is a hallmark of metabolic syndrome [1] and a risk factor of Alzheimer’s disease [2] and vascular dementia [3]. A higher HDL-C level is inversely associated with a lower incidence of cardiovascular disease [4]. Recently, non-HDL-C has been strongly associated with a long-term risk of atherosclerotic cardiovascular disease (CVD) [5]. In addition to the HDL quantity, HDL functionality is also important for protecting against CVD [6] and cerebrovascular disease [2]. These reports raised the importance of both the quantity and quality of HDL to prevent the long-term risk of CVD and cerebrovascular disease [7].

A low HDL-C level in men and women is defined as <40 and <50 mg/dL, respectively, according to the guidelines of the National Cholesterol Education Program-Adult Treatment Panel III. [8]. A previous study reported that Korean women had a 5 mg/dL higher HDL-C level than men from the unadjusted mean difference in HDL-C [9]. On the other hand, no reports explain why women have a higher diagnostic standard of HDL-C than men.

Understanding when and why men have a lower HDL-C level and a shorter life span than women in adulthood and later life is fascinating [10]. In our recent report, the women group had an 8.2 mg/dL higher HDL-C level than the men group in their 20s [11], but the reason for this is still unclear. On the other hand, both groups showed a similar HDL-C level in their 80s: approximately 45.9 and 46.6 mg/dL for men and women, respectively, suggesting that a change in sexual functionality, such as menopause, can decrease the HDL-C level. During the menstrual age, the women group showed a higher HDL-C level, and women live longer than men, but the reason why is unclear [12].

Interestingly, Korean eunuchs live approximately 14.4–19.1 years longer than non-castrated men of a similar socioeconomic status [13]. Furthermore, castrated men residing in a mental hospital lived 14 years longer than intact men in the same hospital [14]. The longer lifespan of eunuchs and castrated men have been associated with less production of male hormones and spermatogenesis [13]. Generally, men have a higher incidence of hypertension than premenopausal women, and it is also linked, at least in part, to a low HDL-C being associated intimately with high blood pressure (BP) [15]. The higher probability of hypertension and dyslipidemia might contribute to the higher mortality of men for decades and all societies [16].

Many questions regarding the gap of the serum HDL-C level and inequality of life expectancy between gender and age have been raised consistently. To answer these unanswered questions, this study compared the HDL-C level during the teenage years, 10 to 19 years of age, from the Korean national health and nutrition examination survey (2013–2017). A recent report showed that a group of women aged 20–29 (58.1 ± 11.3 mg/dL) had higher HDL-C levels (49.9 ± 11.1 mg/dL) in the Seventh Korean National Health and Nutrition Examination Survey in 2017 (KNHANES VII-2) [11]. In addition, there has been no report of a change in the TC, HDL-C, and LDL-C data in teenagers. This study was designed to investigate when and at what age girls had higher HDL-C levels than boys in Korean teenagers.

## 2. Methods

### 2.1. Study Population

This research was based on the sixth and seventh Korean National Health and Nutrition Examination Survey from 2013 to 2017 (KNHANES VI and VII-2, approval number 117002).

The KNHANES is a multistage stratified, complex design survey of a representative sample of the entire Korean population conducted by the Korea Centers for Disease Control and Prevention. Trained interviewers and laboratory technicians conducted surveys in households, including administering questionnaires, performing health examinations, and collecting blood samples.

**Figure 1:**
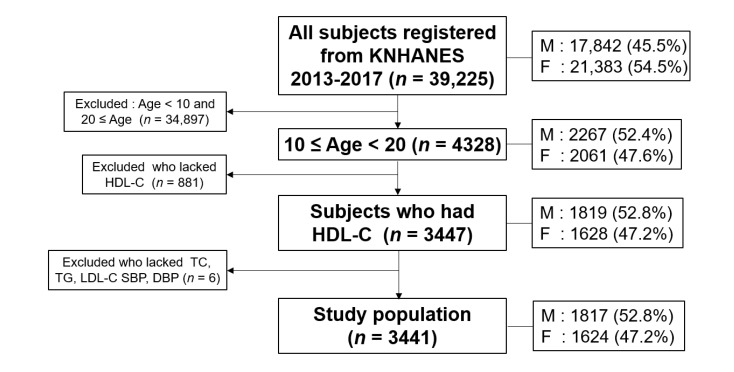
Inclusion criteria and subject number in analysis. HDL-C, high-density lipoprotein cholesterol; TC, total cholesterol; TG, triglycerides; LDL-C, low-density lipoprotein-cholesterol; SBP, systolic blood pressure; DBP, diastolic blood pressure; M: men; F: female.

The total number of KNHANES subjects from 2013 to 2017 was 39,225 individuals from one to 80 years of age, as shown in Figure 1. Among them, the teenager period, 10–19 years of age, in whom blood biochemistry data were available, including HDL-C, total cholesterol (TC), and triglyceride (TG) levels, were selected. Among them, people with data on systolic blood pressure (SBP) and diastolic blood pressure (DBP) were included. Consequently, 3441 (1817 men and 1642 women) subjects were finally selected and analyzed.

The serum TC and HDL-C were measured directly by a homogeneous enzymatic method using Pureauto SCHO-N, Cholest N HDL, and Cholestest LDL agent (Sekisui, Tokyo, Japan) on a Hitachi Automatic Analyzer 7600-210 (Hitachi, Tokyo, Japan). For laboratory measurements, inter-assay coefficient variations (CV) were calculated using the results of internal controls, and intra-assay CVs using data from several pilot studies on both fresh and frozen samples in according to the protocol of lipid standardization program of Center for Disease Control and Prevention (http://www.cdc.gov/nchs/nhanes/2007-2008/HDL_E.htm accessed on 19 October 2021)

### 2.2. Statistical Analysis

Because the KNHANES uses a sophisticated sampling design, the missing values were set to valid values to perform statistical analysis. All analyses were normalized by a homogeneity test of the variances through Levene’s statistics. If not normalized, the nonparametric statistics were analyzed using different methods, such as a Jonckheere-Terpstra test and a Kruskal–Wallis test. All values are expressed as the mean ± SD (standard deviation) for the continuous variables for the lipid and blood pressure changes, as listed in Table 1. The correlation between the lipid and blood pressure changes in Korean teenagers, including the puberty period, were analyzed, as shown in Table 2.

**Figure 2:**
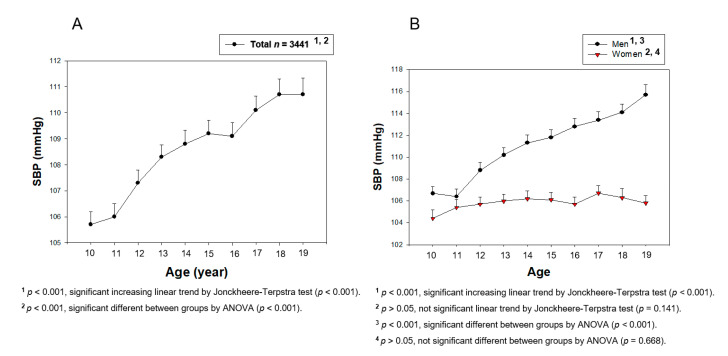
Average systolic blood pressure (SBP) levels by age in total (**A**) or men and women (**B**). Data are mean ± SEM (Standard Error of the Mean).

**Figure 3:**
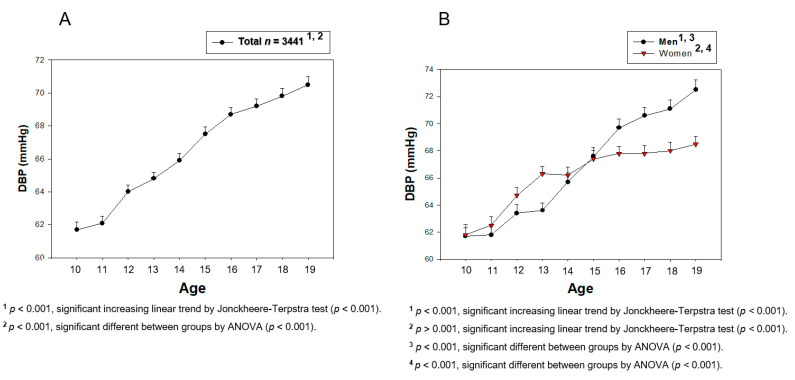
Average of diastolic blood pressure (DBP) levels by age in total (**A**) or men and women (**B**). Data are mean ± SEM (Standard Error of the Mean).

**Figure 4:**
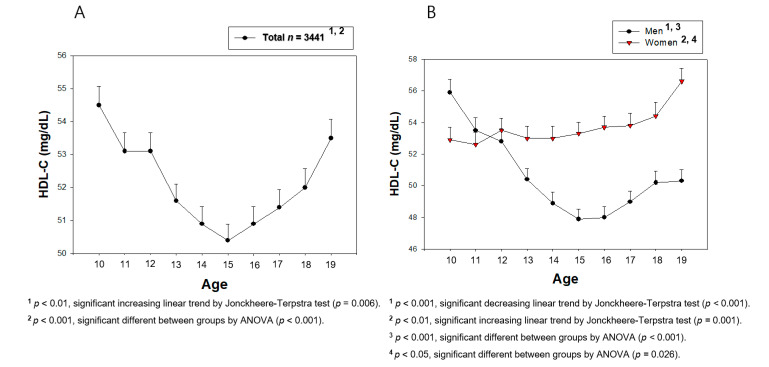
Average of high-density lipoprotein cholesterol (HDL-C) levels by age in total (**A**) or men and women (**B**). Data are mean ± SEM (Standard Error of the Mean).

**Figure 5:**
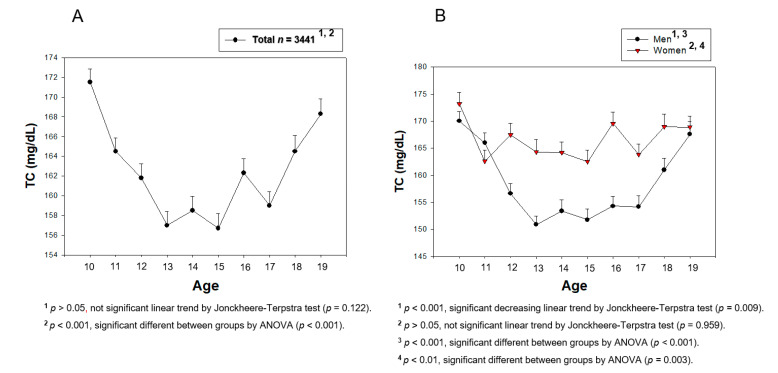
Average of total cholesterol levels (TC) by age in total (**A**) or men and women (**B**). Data are mean ± SEM (Standard Error of the Mean).

**Figure 6:**
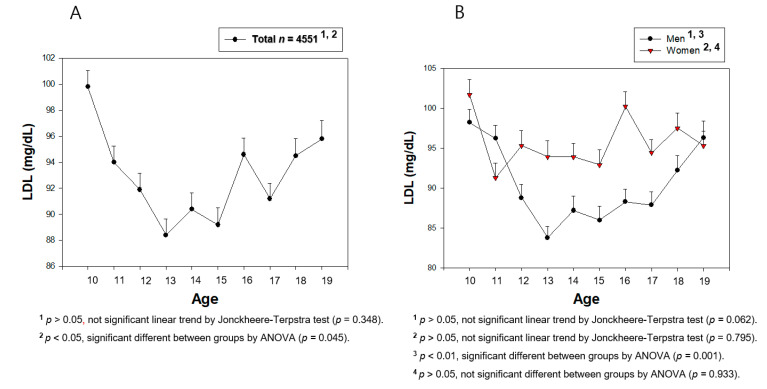
Average of low-density lipoprotein-cholesterol (LDL-C) levels by age in total (**A**) or men and women (**B**). Data are mean ± SEM (Standard Error of the Mean).

**Figure 7:**
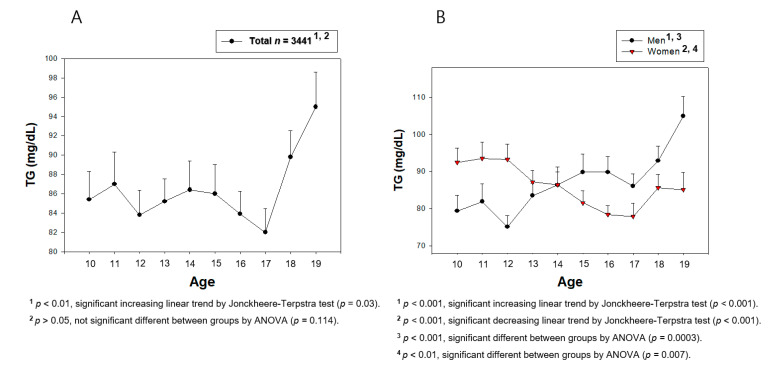
Average triglyceride (TG) levels by age in total (**A**) or men and women (**B**). Data are mean ± SEM (Standard Error of the Mean).

From Figure 2, Figure 3, Figure 4, Figure 5, Figure 6 and Figure 7, the continuous levels of lipid parameters and blood pressure, such as HDL-C, TC, LDL-C, TG, SBP, and DBP, were compared using a Student t-test within each gender depending on age. The differences in the serum concentrations of HDL-C, TC, LDL-C, TG, SBP, and DBP depending on age were determined using ANOVA. A Bonferroni and Games–Howell post-hoc test was used to determine the significance of the differences in the continuous variables and to identify the differences between each age. A multiple group test procedure was performed to determine the pattern, either an increase or decrease in Figs. 2 to 7, according to a Jonckheere–Terpstra test (J-T test), as described previously [17,18].

All tests were two-tailed, and the statistical significance was defined at *p* < 0.05. Statistical analyses were carried out using the SPSS statistical package version 25.0 (SPSS Inc., Chicago, IL, USA), incorporating the sampling weights and adjusting for the sophisticated survey design of the KNHANES 2013-2017.

### 2.3. Ethics Statement

Korea National Health and Nutrition Examination Survey (KNHANES) is an annual review that has been approved by the KCDC Research Ethics Review Committee since 2007 (approval no. 2013–12EXP03–5C). The committee operates under the KCDC Research Ethics Review Committee’s standard guidelines based on domestic & international regulations and guidelines, such as the Declaration of Helsinki and the Bioethics and Safety Act. Informed consent was obtained from all participants when the surveys were conducted.

## 3. Results

### 3.1. Change in BP during Teenage

As shown in Table 1 and Figure 2A, the total population showed a gradual and steady increase in the SBP from 10 (105.7 ± 8.8 mmHg) to 19 years of age 110.7 ± 11.5 mmHg, *p* < 0.001). On the other hand, there was a difference between boys and girls of the same age (*p* = 0.024, Figure 2B). The boys’ group showed a more distinct and sharper increase in the SBP from 10 (106.7 ± 8.3 mmHg) to 19 years of age (115.7 ± 11.9 mmHg, *p* < 0.001), whereas the girls’ group showed a similar SBP from 10 (104.4 ± 9.3 mmHg) to 19 years of age (105.8 ± 8.6 mmHg, *p* <0.141). The boys’ group showed a nine mmHg difference (*p* < 0.001) between 10 and 19 years of age, whereas the girls’ group did not show a difference among any age interval (*p* = 0.668 from ANOVA).

Correlation analysis showed that the boys’ group only showed positive correlations (r *=* 0.277, *p* < 0.001) between 10 and 19 years of age (Table 2 and Figure S1A), but the girls’ group did not show any significance (r *=* 0.041, *p* < 0.101). The boys’ group showed higher positive correlations (r *=* 0.208, *p* < 0.001) between 10 and 15 years of age (Figure S1B) compared to that between 15 and 19 years of age (r *=* 0.122, *p* = 0.0003, Figure S1C). These results suggest that age has a stronger influence on the SBP in the boys’ group, which increased consistently until 19 years of age, than the girls’ group.

Interestingly, both the boys’ and girls’ groups showed a similar DBP at 10 years of age (~61.7 mmHg) and a gradual increase in the DBP during the teenage period (boys and girls, *p* < 0.001), as shown in Table 1 and Figure 3B. The boys’ group had a higher DBP (72.5 ± 9.3 mmHg) than the girls’ group (68.5 ± 7.4 mmHg) at 19 years of age (*p* *=* 0.00002). Correlation analysis showed that both the boys (r *=* 0.406, *p* < 0.001) and girls’ groups (r *=* 0.254, *p* < 0.001) had positive correlations between 10 and 19 years of age (Table 2 and Figure S2A). Interestingly, the boys’ and girls’ group showed similar positive correlations (r *=* 0.233 and 0.239, *p* < 0.001) between 10 and 15 years of age (Figure S2B). On the other hand, the girls’ group did not show positive correlations (r *=* 0.046, *p* = 0.186) between 15 and 19 years of age, whereas the boys’ group still showed a positive correlation (r *=* 0.183, *p* < 0.001, Figure S1C). In addition, the DBP in the boys’ group also showed a stronger correlation (r *=* 0.406) than the girls’ group. At 19 years of age, the boys’ group showed a higher SBP and DBP than the girls’ group, suggesting there was a different pattern of increase between the two groups.

### 3.2. Change in the HDL-C Level during the Teenage Period

The total population in the teenage period (*n* = 3,441) showed a sharp decrease in the HDL-C level from 10 (54.5 ± 10.2 mg/dL) to 15 years of age (50.4 ± 9.4 mg/dL, *p* < 0.001), followed by an increase to 19 years of age (53.5 ± 10.4 mg/dL, *p* = 0.0001). Still, the final level was lower than the initial level at 10 years of age (Figure 4A) with a resulting U-shaped pattern of HDL-C level versus age. As shown in Table 1 and Figure 4B, the boys’ group showed a sharper decrease in the HDL-C level from 10 (55.9 ± 10.6 mg/dL, *p* < 0.001) to 15 years of age (47.9 ± 8.7 mg/dL), followed by a slow increased to 19 years of age (50.3 ± 9.2 mg/dL, *p* = 0.001). The girls’ group did not show a decrease in the HDL-C level from 10 (52.9 ± 9.5 mg/dL) to 19 years of age (56.6 ± 10.6 mg/Dl, *p* = 0.001), but the HDL-C increased gradually during the same period. Interestingly, the boys’ group showed a 3.0 mg/dL higher HDL-C level (*p* = 0.011) than the girls’ group at 10 years of age, but the boys’ group showed a 6.3 mg/dL lower HDL-C level than the girls’ group at 19 years of age (*p* < 0.001). These results strongly suggest a significantly different pattern of HDL-C changes between the two groups (*p* < 0.001 for boys; *p* = 0.026 for girls).

Correlation analysis showed that the boys’ group had a negative correlation (r *=* −0171, *p* < 0.001) between the increase in age and HDL-C between 10 and 19 years of age (Table 2 and Figure S3A), whereas the girls’ group showed a positive correlation (r *=* 0.085, *p* = 0.0006). In the early pubertal period, between 10 and 15 years of age, only the boys’ group showed a more negative association (r *=* −0.256, *p* < 0.001), where the HDL-C level decreased with age, whereas the girls’ group did not show a significant change (r *=* 0.011, *p* = 0.723, Figure S3B). From 15 to 19 years of age, both the boys’ group (r *=* 0.111, *p* = 0.001) and girls’ group (r *=* 0.104, *p* = 0.003) showed positive correlations between the HDL-C level and age (Figure S3C).

### 3.3. Change in the Serum TC during the Teenage Period

As shown in Figure 5A and Table 1, the total population showed a rapid decrease in the TC level from 10 to 13 years of age, suggesting that the demand for cholesterol for growth was elevated. The TC level then increased slowly until 19 years of age, resulting in a U-shaped pattern of TC depending on the increase in age. The serum TC level decreased more sharply from 10 (170.0 ± 23.0 mg/dL) to 15 years of age (151.8 ± 27.3 mg/dL, *p* < 0.001) in the boys’ group, and then increased slowly again to 167.6 ± 29.0 mg/dL at 19 years of age (*p* < 0.001). The lowest point of TC was 13 years of age (150.9 ± 24.1 mg/dL) in the boys’ group. On the other hand, the girls’ group showed a decreasing pattern from 10 to 15 years of age (*p* = 0.003) from 173 ± 25.3 mg/dL to 162.5 ± 27.6 mg/dL, but they showed no change in the TC level from 15 to 19 years of age (*p* = 0.065). The girls’ group showed no change in the TC level between 10 and 19 years of age (*p* = 0.959).

Figure S4A shows that there was no correlation between the increase in age and the change in the TC level in both the boys’ group and girls’ group from 10 to 19 years of age. On the other hand, both groups showed a negative correlation between 10 and 15 years of age, but the boys (r *=* −0.244, *p* < 0.001) group showed a three-fold higher negative correlation than the girls’ group (r *=* −0.088, *p* = 0.006). Between 15 and 19 years of age, only the boys’ group showed positive correlations (r *=* 0.194, *p* < 0.001), whereas the girls’ group showed weak positive correlations without significance between the TC level and age. In the total population, there was no correlation between the TC level and age between 10 and 19 years of age because the gap of the TC level depending on age was too large. In the boys’ group, however, there was a distinct negative and positive correlation between 10 and 15 years of age and between 15 and 19 years of age, respectively.

### 3.4. Change in the Serum LDL-C during Teenage

As shown in Figure 6A and Table 1, the serum LDL-C level was decreased sharply from 10 (99.8 ± 21.9 mg/dL) to 13 years of age (88.4 ± 24.7 mg/dL, *p* < 0.001), and then increased to 95.8 ± 25.1 mg/dL at 19 years of age (*p* < 0.001). The boys’ group showed a sharp decrease from 10 (98.2 ± 21.6 mg/dL) to 13 years of age (83.8 ± 21.3 mg/dL, *p* < 0.001), and then a slow increase to 96.3 ± 26.9 mg/dL at 19 years of age (*p* = 0.00008, Figure 6B). As with the TC level, the change in the LDL-C level in the boys’ group also showed a U-shaped pattern during the teenage years. The girls’ group showed a decreasing pattern between 10 and 15 years of age (*p* = 0.043), and no notable change between 15 and 19 years of age (*p* = 0.596).

Between 10 and 19 years of age, both groups did not show any correlations between the LDL-C and the increase in age (Figure S5A). In the early pubertal age, the boys’ group showed a strong negative correlation (r *=* −0.192, *p* < 0.001) between 10 and 15 years of age (Figure S5B), and then a positive correlation (r *=* 0.143, *p* = 0.00002) between 15 and 19 years of age (Figure S5C). The girls’ group showed a negative correlation (r *=* −0.071, *p* = 0.027) only between 10 and 15 years of age. In the total population, there was no correlation between the LDL-C level and age between 10 and 19 years because the gap of the TC depending on each age was too large. On the other hand, particularly in the boys’ group, there was a distinct negative and positive correlation between 10 and 15 years of age and between 15 and 19 years of age, respectively.

### 3.5. Change in the Serum TG during the Teenage Years

The total population showed an increase in the serum TG level, especially in the late teenage years, 18 to 19 years of age, as shown in Figure 7A. The highest TG (95.0 ± 64.2 mg/dL) level was observed in the 19 years of age group of the total population. Interestingly, the boys’ group showed a 13.1 mg/dL lower TG level at 10 years of age (*p* = 0.027) and a 19.9 mg/dL higher TG level at 19 years of age (*p* = 0.005) than the girls’ group (Figure 7B). The girls’ group showed a generally decreasing pattern of the TG level between 10 and 17 years of age. In contrast, the boys’ group showed a gradually increase pattern between 10 and 19 years of age (*p* < 0.001), but both groups showed a significant deviation. Depending on age, the boys’ group showed an increase in the serum TG level, whereas the girls’ group showed a decrease in the TG level between 10 and 19 years of age.

From 10 to 19 years of age, correlation analysis revealed a positive correlation between the TG level and age in the boys’ group (r = 0.111, *p* = 0.000002), but a negative correlation in the girls’ group (r = −0.086, *p* = 0.0005), as shown in Figure S6A. The boys’ group showed a constant positive correlation between 10 and 15 years of age (r = 0.061, *p* = 0.041) and 15 and 19 years of age (r = 0.078, *p* = 0.022), as shown in Figure S6B and 6C. On the other hand, the girls’ group showed a negative correlation (r = −0.086, *p* = 0.008) only between 10 and 15 years of age.

### 3.6. Correlation Analysis between the HDL-C Level and BP

Between 10 and 19 years of age, the boys’ group showed a two times higher negative correlation (r *=* −0.133, *p* < 0.001) between the HDL-C level and the SBP than the girls’ group (r *=* −0.065, *p* = 0.009), as shown in Table 2 and Figure S7A. In particular, in the early pubertal age (10–15 years of age), only the boys’ group showed a significant negative correlation (r *=* −0.115, *p* = 0.0001) (Figure S7B). In the late pubertal age (15–19 years of age), the boys’ group still showed a higher negative correlation (r *=* −0.100, *p* = 0.003) than the girls’ group (r *=* −0.074, *p* = 0.034), as shown in Figure S7C. Overall, these results clearly showed an inverse correlation between the HDL-C level and BP; the HDL-C decreased with increasing BP.

Interestingly, between 10 and 19 years of age, the boys’ group showed a 7.6-fold higher negative correlation (r *=* −0.122, *p* < 0.001) between the HDL-C level and the DBP than the girls’ group (r *=* −0.016, *p* *=* 0.516), as shown in Table 2 and Figure S8A. In the early and late pubertal age, only the boys’ group showed a significant negative correlation between the HDL-C level and the DBP (Table 2, Figure S8B,C), suggesting that a higher HDL-C level is correlated with a lower DBP only in the boys’ group.

## 4. Discussion

Why and when men have a lower HDL-C level than women in adulthood has been an unanswered question for a long time. To help answer this question, this study compared the HDL-C levels of teenage subjects (*n* = 3441), 10 to 19 years of age, from 2013 to 2017 in the KNHANES VI and VII-2. The current study revealed a change in the serum lipid profile, particularly the HDL-C level, and its correlation with BP during the teenage period. The major findings are that the BP increased gradually (Figure 2 and Figure 3), and the HDL-C level decreased rapidly in the pubertal period in the boys’ group (Table 1 and Figure 4). The TC and LDL-C levels also decreased in the pubertal period at 15 years of age in both groups, but it was increased to the initial level at 19 years of age. The boys’ group showed a similar TC and LDL-C level to those of the girls’ group at 19 years of age. On the other hand, the lower HDL-C level at 15 years of age was not restored at 19 years of age in the boys’ group; it was almost fixed and remained at a lower level for the lifetime of men compared to that of women.

These results strongly suggest that the HDL-C level at 15 years of age in the boys’ group was a critical event to explain why the men group has a lower HDL-C level than the women group at the same age in adulthood until 50 years of age. Between 10 to 15 years of age, the boys’ group showed a distinct decreasing pattern (*p* < 0.001), but the girls’ group showed no change (*p* = 0.588). Furthermore, the boys’ group showed a decreasing tendency between 10 and 19 years of age (*p* < 0.001), but the girls’ group showed an increasing tendency (*p* = 0.001). Interestingly, the girls’ group also showed a decrease in the TC (*p* = 0.003) and LDL-C (*p* = 0.043) between 10 and 15 years of age, similar to the boys’ group (*p* for TC and LDL-C <0.001, Table 1). In contrast to the girls’ group, these results strongly suggest that a decrease in the HDL-C in the pubertal age occurred only in the boys’ group, but the reason for this is unclear. One possible explanation is the consumption of cholesterol from HDL for the production of the male hormone and spermatogenesis in boys. The plasma HDL-C level was reported to be at the lowest level and was inversely related to the testosterone level at pubertal age (around 15 years of age). Cholesterol plays a crucial role in spermatogenesis and steroidogenesis in the male reproduction system. Sertoli cells, which promote sperm production, block the passage of LDL at the blood–testis barrier (BTB) but permit the entry of HDL to the seminiferous tubules [19]. Cholesterol is essential for spermatogenesis because it serves as a fuel for Sertoli cells. In addition to the blood–brain barrier (BBB), it is fascinating that HDL can cross the BTB, whereas LDL cannot cross both barriers. This is the dramatic difference between males and females that can help explain why in boys, the HDL-C was lower since the puberty period due to spermatogenesis. Recently, it was proposed that blood-originated HDL could cross the BBB to inhibit amyloidogenesis and deliver amyloid back to the blood for removal [20].

The puberty period is critical to differentiating sexual functionality in both males and females, and this period is accompanied by hormonal changes and cholesterol requirements. On the other hand, men and women have different patterns in consuming cholesterol for sexual development and the reproduction system. The uptake of cholesterol in Sertoli cells is strongly dependent on scavenger receptor B-I (SR-BI), which is for the HDL-docking receptor [21]. In the rodent model, cholesterol from HDL served as the primary source of cholesterol for Sertoli cells [22]. Although the reason for this is still unclear, only cholesterol from HDL should be used as a fuel for spermatogenesis in the Sertoli cells. A possible answer is that the relatively smaller size of HDL with the antioxidant ability and anti-inflammatory activity can protect spermatids and spermatogonia. Similarly, in the same context, treatment with NaCl caused the degradation of HDL, and the high dose consumption of NaCl in zebrafish resulted in an impairment of testicular spermatogenesis and male infertility [23]. Overall, these reports highlight the importance of HDL for protecting the male reproduction system and supplying cholesterol during spermatogenesis. Eunuchs lived longer than uncastrated men from the same social class in the Chosun dynasty, a few hundred years ago in Korea [13]. In addition, earlier castration, before 8–14 years of age, was associated with a longer life expectancy of approximately 76.3 years, whereas the non-castrated control showed a life expectancy of 64.7 years [14].

A longitudinal study with 633 participants of Americans, including African Americans, aged 8–18 years, showed that the HDL-C level decreased during puberty in the male group, whereas the female group showed no change [24]. Another longitudinal study with Japanese participants, 1442 boys and 1350 girls, also showed a decrease in the HDL-C level in only the boys’ group between 10 and 14 years of age. In contrast, the girls’ group did not show a change in the HDL-C during the same period. An Italian study also showed that the boys’ group had a lower TC level than the girls’ group at 14 to 15 years of age, even though both groups had a similar TC level at 11 years of age [25]. Before the pubertal age, at 10 years of age, the boys’ group showed a significantly higher HDL-C level than the girls’ group with the same DBP. On the other hand, the boys’ group showed a rapid decrease in the HDL-C level during the pubertal period, 13 to 16 years of age. In many mammals, including humans and apes, females consistently live longer than males [26]. Women tend to live longer than men. The average American man expects to live to 76 years of age, whereas the average woman in America expects to live to 81 years of age [27]. One possible explanation might be that the lowered HDL-C level is associated with a higher prevalence of hypertension [28] and cardiovascular events, and a lower HDL-C pattern contributes to men’s elevated rates of coronary heart disease and mortality [29]. Quantity and quality of HDL are very important to suppress the incidence of metabolic disease and they could be impaired by ageing and an unhealthy lifestyle [30]. As well as HDL quantity, HDL functionality can be enhanced by lifestyle modification and nutritional supplementation [31]. It might be necessary to supplement functional food to boys in puberty period to improve HDL quantity and functionality.

However, the current study has limitation due to the relatively small population of teenagers (*n* = 36,441) in Korean ethnicity. Future studies should be carried out with more populations and in countries. In addition, changes in the composition and functionality in teenagers should be investigated because the current study evaluated only the amount of cholesterol in HDL and LDL.

## 5. Conclusions

The boys’ group has a high demand for cholesterol, particularly HDL, for spermatogenesis during the pubertal period, resulting in a lower HDL-C in adulthood. The lower HDL-C during the teenage period in males may be associated with a lower life expectancy of men in adulthood.

## Figures and Tables

**Table 1:** Trend analysis of the lipid and blood pressure changes during the pubertal period in Korean teenagers.

	Boys (*n* = 1817, mean ± SD)			Girls (*n* = 1624, mean ± SD)		
	10–11 (*n* = 367)	14–15 (*n* *=* 375)	18–19 (*n* *=* 333)	10–11 (*n* *=* 294)	14–15 (*n* *=* 332)	18–19 (*n* *=* 296)
HDL-C (mg/dL)	54.6 ± 11.1	48.4 ± 9.0	50.3 ± 9.3	52.8 ± 9.6	53.1 ± 9.7	55.6 ± 10.5
*p* for trend	10–15, decrease, <0.001 15–19, increase, 0.001 10–19, decrease, <0.001			10–15, no change, 0.588 15–19, increase, 0.012 10–19, increase, <0.001		
TC (mg/dL)	167.9 ± 24.8	152.6 ± 27.4	164.2 ± 28.8	167.7 ± 25.7	163.3 ± 26.4	168.9 ± 26.2
*p* for trend	10–15, decrease, <0.001 15–19, increase, <0.001 10–19, decrease, 0.009			10–15, decrease, 0.003 15–19, no change, 0.065 10–19, no change, 0.959		
LDL-C (mg/dL)	97.1 ± 22.5	86.6 ± 24.4	94.2 ± 25.7	96.3 ± 23.0	93.4 ± 23.1	96.3 ± 22.5
*p* for trend	10–15, decrease, <0.001 15–19, increase, 0.00008 10–19, no change, 0.062			10–15, decrease, 0.043 15–19, no change, 0.596 10–19, no change, 0.795		
SBP (mmHg)	106.5 ± 9.0	111.6 ± 9.9	114.9 ± 11.0	105.0 ± 9.3	106.2 ± 8.9	106.0 ± 9.0
*p* for trend	10–19, increase, <0.001			10–19, no change, 0.141		
DBP (mmHg)	61.7 ± 8.2	66.7 ± 9.0	71.8 ± 9.0	62.2 ± 8.4	66.8 ± 7.8	68.3 ± 7.4
*p* for trend	10–19, increase, <0.001			10–19, increase, <0.001		

Data are expressed as mean ± SD (standard deviation). HDL-C, high-density lipoprotein-cholesterol; TC, total cholesterol; LDL-C, low-density lipoprotein-cholesterol; SBP, systolic blood pressure; DBP, diastolic blood pressure.

**Table 2:** Correlation analysis of the lipid and blood pressure changes during the pubertal period in Korean teenagers.

		Age	Height	Weight	HDL-C	TC	LDL-C	TG	SBP
Boys (*n* = 1817)	Height (cm)	0.773 ***							
	Weight (kg)	0.596 ***	0.718 ***						
	HDL-C (mg/dL)	−0.171 ***	−0.250 ***	−0.355 ***					
	TC (mg/dL)	−0.042	−0.193 ***	0.052 *	0.269 ***				
	LDL-C (mg/dL)	-0.029	−0.150 ***	0.097 ***	0.053 *	0.888 ***			
	TG (mg/dL)	0.111 ***	0.075 **	0.231 ***	−0.356 ***	0.277 ***	−0.023		
	SBP (mmHg)	0.277 ***	0.288 ***	0.451 ***	−0.133 ***	0.120 ***	0.101 ***	0.190 ***	
	DBP (mmHg)	0.406 ***	0.339 ***	0.329 ***	−0.122 ***	0.061 **	0.054 *	0.140 ***	0.451 ***
Girls (*n* = 1624)	Height (cm)	0.575 ***							
	Weight (kg)	0.488 ***	0.653 ***						
	HDL-C (mg/dL)	0.085 ***	0.014	−0.165 ***					
	TC (mg/dL)	0.004	−0.080 *	0.025	0.293 ***				
	LDL-C (mg/dL)	0.003	−0.070 **	0.064 **	0.052 *	0.914 ***			
	TG (mg/dL)	−0.086 ***	−0.067 **	0.086 ***	−0.354 ***	0.253 ***	0.043		
	SBP (mmHg)	0.041	0.107 ***	0.266 ***	−0.065 **	0.015	0.005	0.102 ***	
	DBP (mmHg)	0.254 ***	0.249 ***	0.290 ***	−0.016	0.003	−0.001	0.027	0.482 ***

*: *p* < 0.05, **: *p* < 0.01, ***: *p* < 0.001. HDL-C, high-density lipoprotein-cholesterol; TC, total cholesterol; LDL-C, low-density lipoprotein-cholesterol; TG, triglyceride; SBP, systolic blood pressure; DBP, diastolic blood pressure.

## Data Availability

The data used to support the findings of this study are available from the corresponding author upon request.

## Supplementary Materials

The following are available online at https://www.mdpi.com/article/10.3390/medsci9020035/s1, Figure S1. Pearson’s correlation analysis of SBP and age in 10–19 years old (A) or 10–15 years old (B) or 15–19 years old (C). Figure S2. Pearson’s correlation analysis of DBP and age in 10–19 years old (A) or 10–15 years old (B) or 15–19 years old (C). Figure S3. Pearson’s correlation analysis of HDL-C and age in 10–19 years old (A) or 10–15 years old (B) or 15–19 years old (C). Figure S4. Pearson’s correlation analysis of TC and age in 10–19 years old (A) or 10–15 years old (B) or 15–19 years old (C). Figure S5. Pearson’s correlation analysis of LDL-C and age in 10–19 years old (A) or 10–15 years old (B) or 15–19 years old (C). Figure S6. Pearson’s correlation analysis of TG and age in 10–19 years old (A) or 10–15 years old (B) or 15–19 years old (C). Figure S7. Pearson’s correlation analysis of HDL-C and SBP in 10–19 years old (A) or 10–15 years old (B) or 15–19 years old (C). Figure S8. Pearson’s correlation analysis of HDL-C and DBP in 10–19 years old (A) or 10–15 years old (B) or 15–19 years old (C).
