# Supplementary material for: Rapid Decrease in HDL-C in the Puberty Period of Boys Associated with an Elevation of Blood Pressure and Dyslipidemia in Korean Teenagers: An Explanation of Why and When Men Have Lower HDL-C Levels Than Women
Source: Med Sci (Basel). 2021 May 24;9(2):35. doi: 10.3390/medsci9020035 (PMC8163168; doi:10.3390/medsci9020035)
Supplement: Supplementary file 1 [file medsci-09-00035-s001.zip › medsci-1202250-supplementary.pdf]

medical sciences

MDPI

Article

Rapid Decrease in HDL-C in the Puberty Period of Boys Associated with an Elevation of Blood Pressure and Dyslipidemia in Korean Teenagers: An Explanation of Why and When Men Have Lower HDL-C Levels than Women

Kyung-Hyun Cho [1,2,3,*] and Jae-Ryong Kim [3]

[1] Korea Research Institute of Lipoproteins, Medical Innovation Complex, Daegu 41061, Korea
[2] LipoLab, Yeungnam University, Gyeongsan 38541, Korea
[3] Department of Biochemistry and Molecular Biology, Smart-Aging Convergence Research Center, College of Medicine, Yeungnam University, Daegu 42415, Korea; kimjr@med.yu.ac.kr
* Correspondence: chok@yu.ac.kr; Tel.: +82-53-964-1990; Fax: +82-53-965-1992

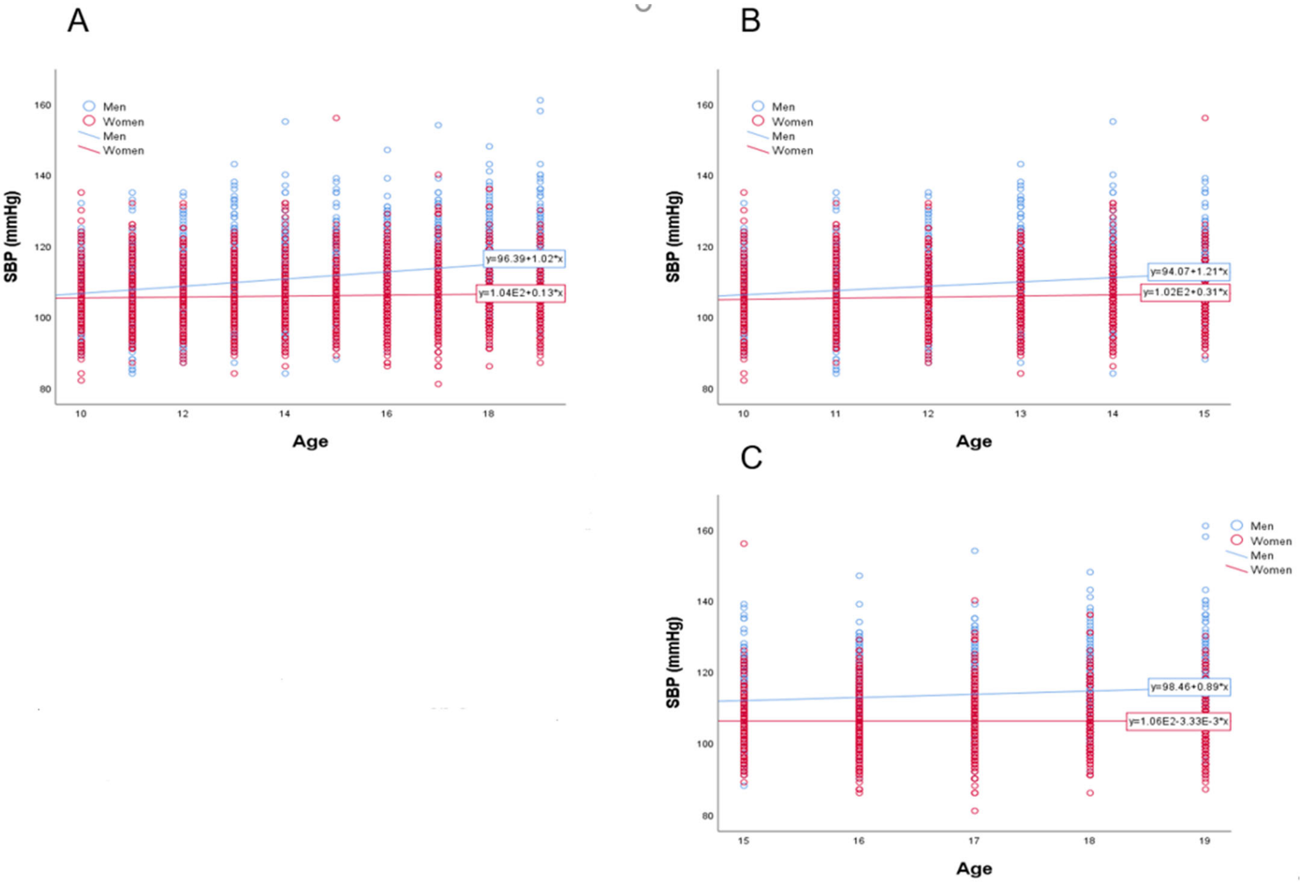

Figure S1. Pearson's correlation analysis of SBP and age in 10–19 years old (A) or 10–15 years old (B) or 15–19 years old (C). In panel A (10–19 years-old) the men group showed positive correlation (r = 0.277, $p < 0.001$) and the women group showed no correlation (r = 0.041, $p = 0.101$). In panel B (10–15 years-old) the men group showed positive correlation (r = 0.208, $p < 0.001$) and the women group showed no correlation (r = 0.059, $p = 0.068$). In panel C (15–19 years-old) the men group showed positive correlation (r = 0.122, $p = 0.0003$) and the women group showed no correlation (r = −0.001, $p = 0.988$)

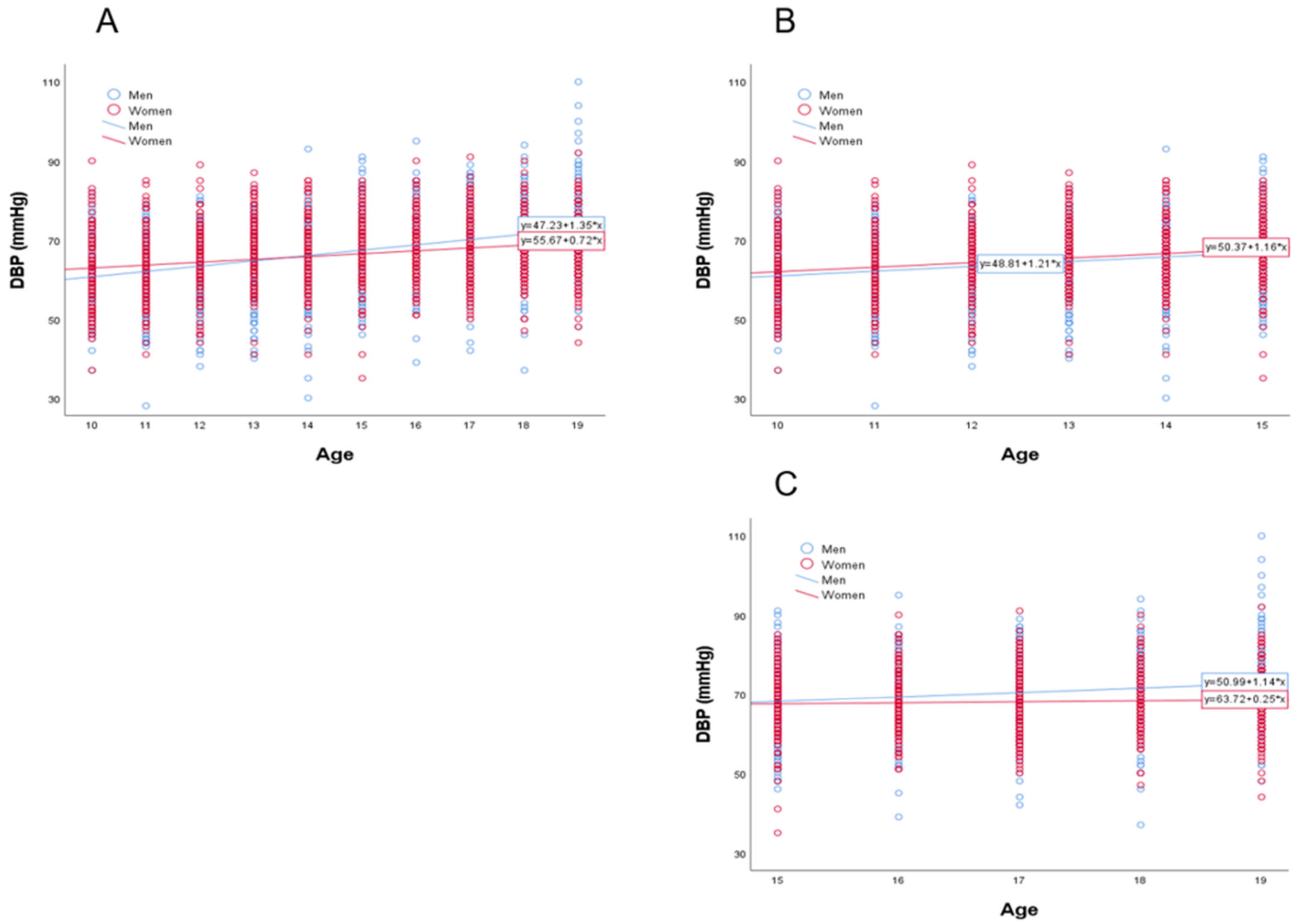

Figure S2. Pearson's correlation analysis of DBP and age in 10–19 years old (A) or 10–15 years old (B) or 15–19 years old (C). In panel A (10–19 years-old) the men group showed positive correlation (r = 0.406, $p < 0.001$) and the women group showed positive correlation (r = 0.254, $p < 0.001$). In panel B (10–15 years-old) the men group showed positive correlation (r = 0.233, $p < 0.001$) and the women group showed positive correlation (r = 0.239, $p < 0.001$). In panel C (15–19 years-old) the men group showed positive correlation (r = 0.183, $p < 0.001$) and the women group showed no correlation (r = −0.046, $p = 0.186$).

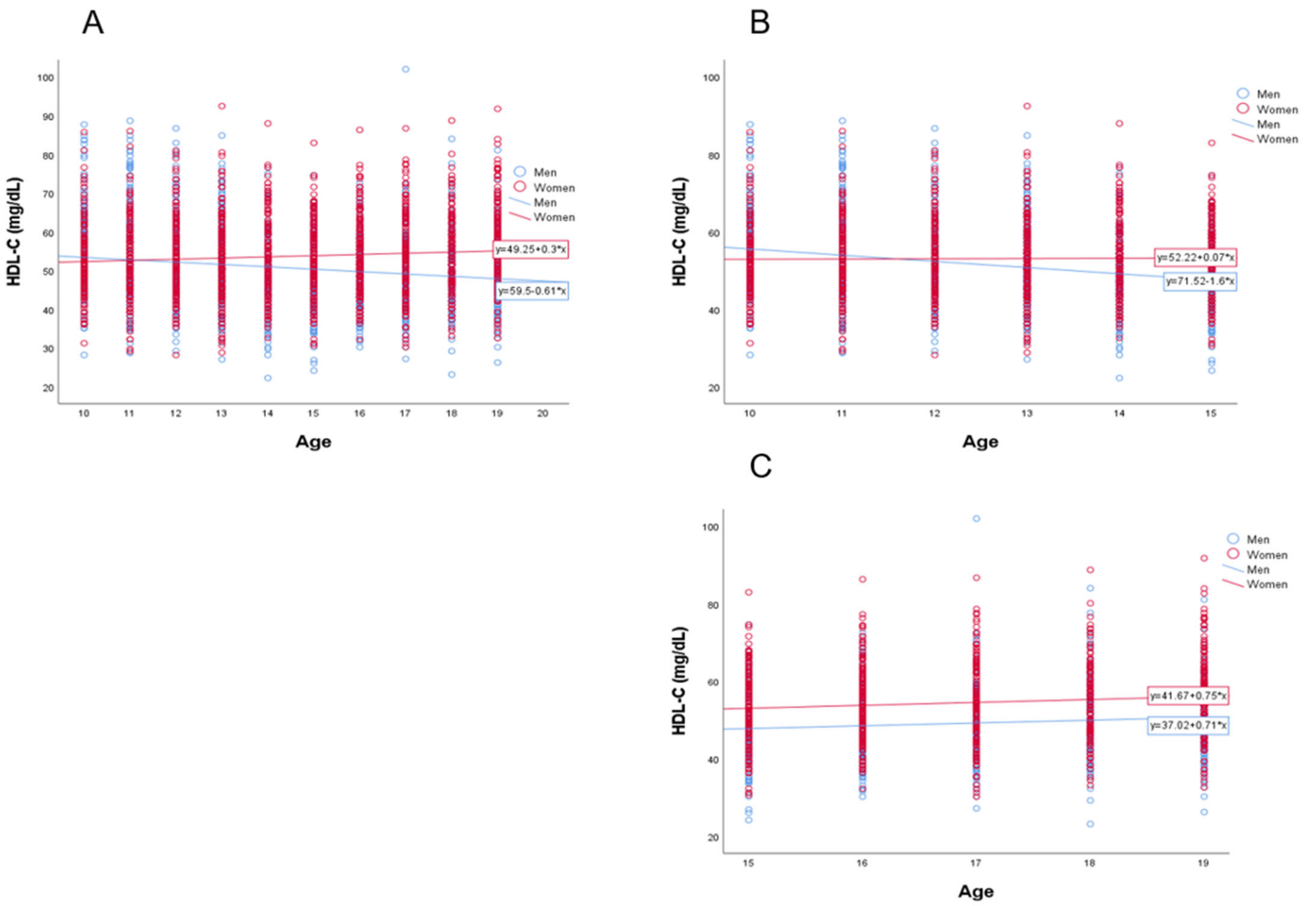

Figure S3. Pearson's correlation analysis of HDL-C and age in 10–19 years old (A) or 10–15 years old (B) or 15–19 years old (C). In panel A (10–19 years-old) the men group showed negative correlation (r = −0.171, $p < 0.001$) and the women group showed positive correlation (r = 0.085, $p = 0.0006$). In panel B (10–15 years-old) the men group showed negative correlation (r = −0.256, $p < 0.001$) and the women group showed no correlation (r = 0.011, $p = 0.723$). In panel C (15–19 years-old) the men group showed positive correlation (r = 0.111, $p = 0.001$) and the women group showed positive correlation (r = 0.104, $p = 0.003$).

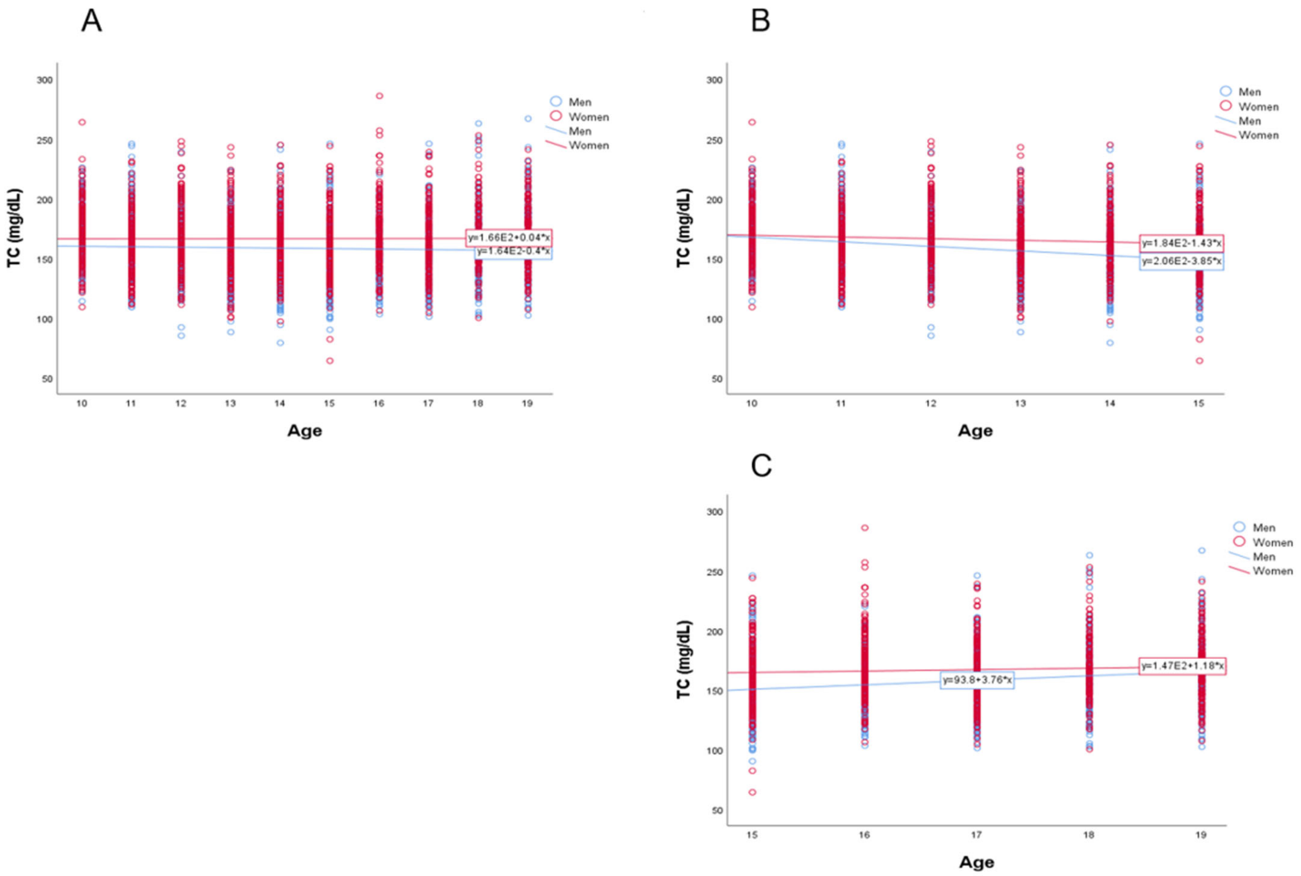

Figure S4. Pearson's correlation analysis of TC and age in 10–19 years old (A) or 10–15 years old (B) or 15–19 years old (C). In panel A (10–19 years-old) the men group showed no correlation (r = −0.042, *p* = 0.076) and the women group showed no correlation (r = 0.004, *p* = 0.871). In panel B (10–15 years-old) the men group showed negative correlation (r = −0.244, *p* < 0.001) and the women group showed negative correlation (r = −0.088, *p* = 0.006). In panel C (15–19 years-old) the men group showed positive correlation (r = 0.194, *p* < 0.001) and the women group showed no correlation (r = 0.062, *p* = 0.078).

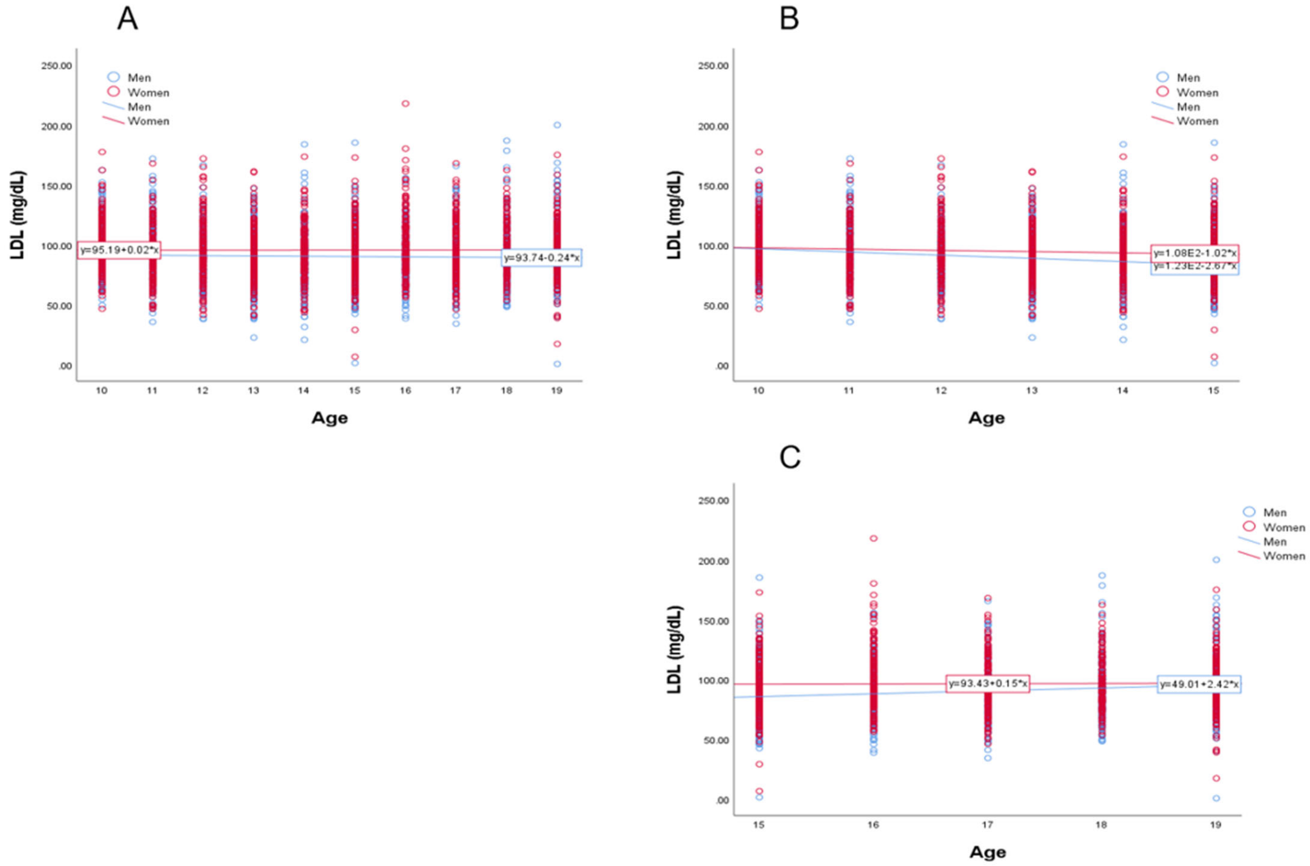

Figure S5. Pearson's correlation analysis of LDL-C and age in 10–19 years old (A) or 10–15 years old (B) or 15–19 years old (C). In panel A (10–19 years-old) the men group showed no correlation (r = −0.029, $p = 0.219$) and the women group showed no correlation (r = 0.003, $p = 0.908$). In panel B (10–15 years-old) the men group showed negative correlation (r = −0.192, $p < 0.001$) and the women group showed negative correlation (r = −0.071, $p = 0.027$). In panel C (15–19 years-old) the men group showed positive correlation (r = 0.143, $p = 0.00002$) and the women group showed no correlation (r = 0.009, $p = 0.792$).

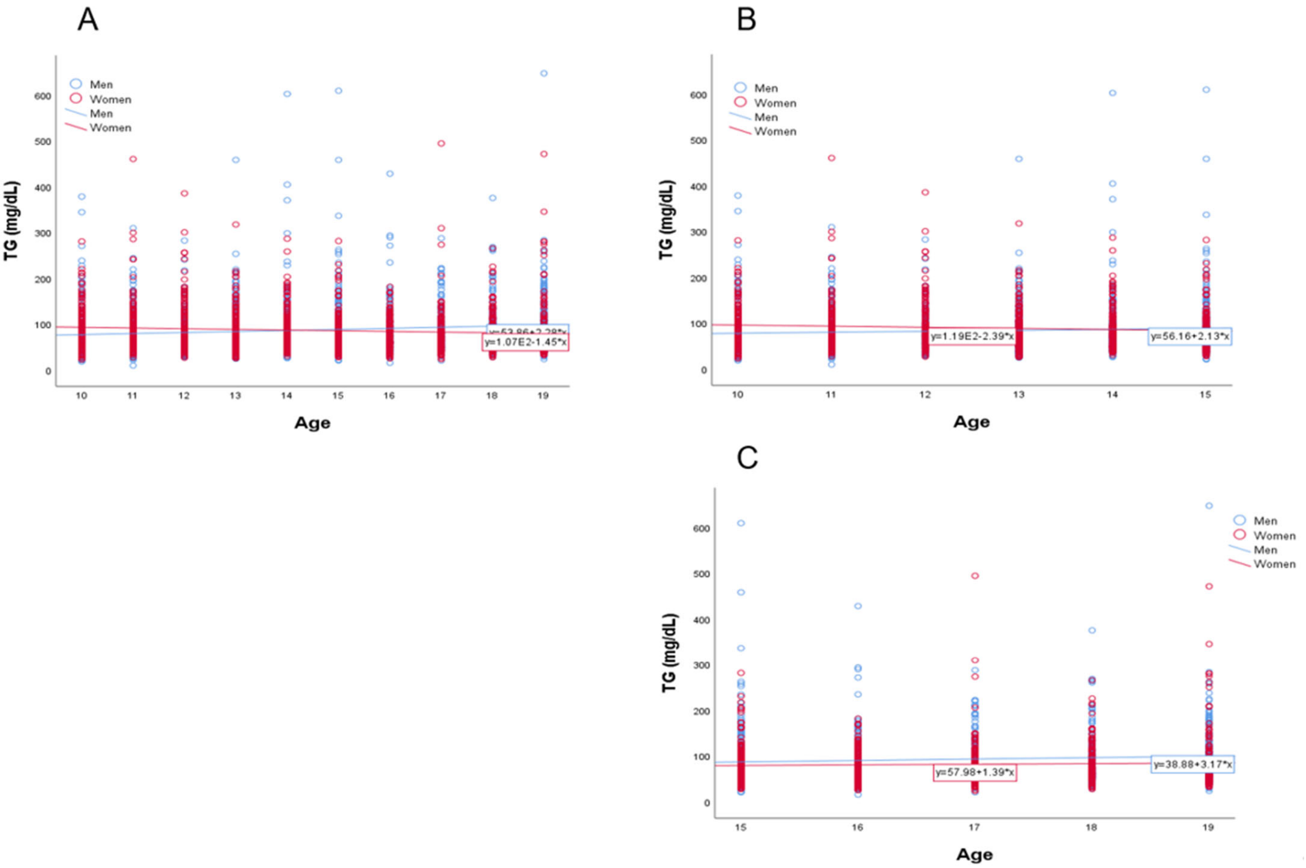

Figure S6. Pearson's correlation analysis of TG and age in 10–19 years old (A) or 10–15 years old (B) or 15–19 years old (C). In panel A (10–19 years-old) the men group showed positive correlation (r = 0.111, p = 0.000002) and the women group showed negative correlation (r = −0.086, p = 0.0005). In panel B (10–15 years-old) the men group showed positive correlation (r = 0.061, p < 0.041) and the women group showed negative correlation (r = −0.086, p = 0.008). In panel C (15–19 years-old) the men group showed positive correlation (r = 0.078, p = 0.022) and the women group showed no correlation (r = 0.043, p = 0.223).

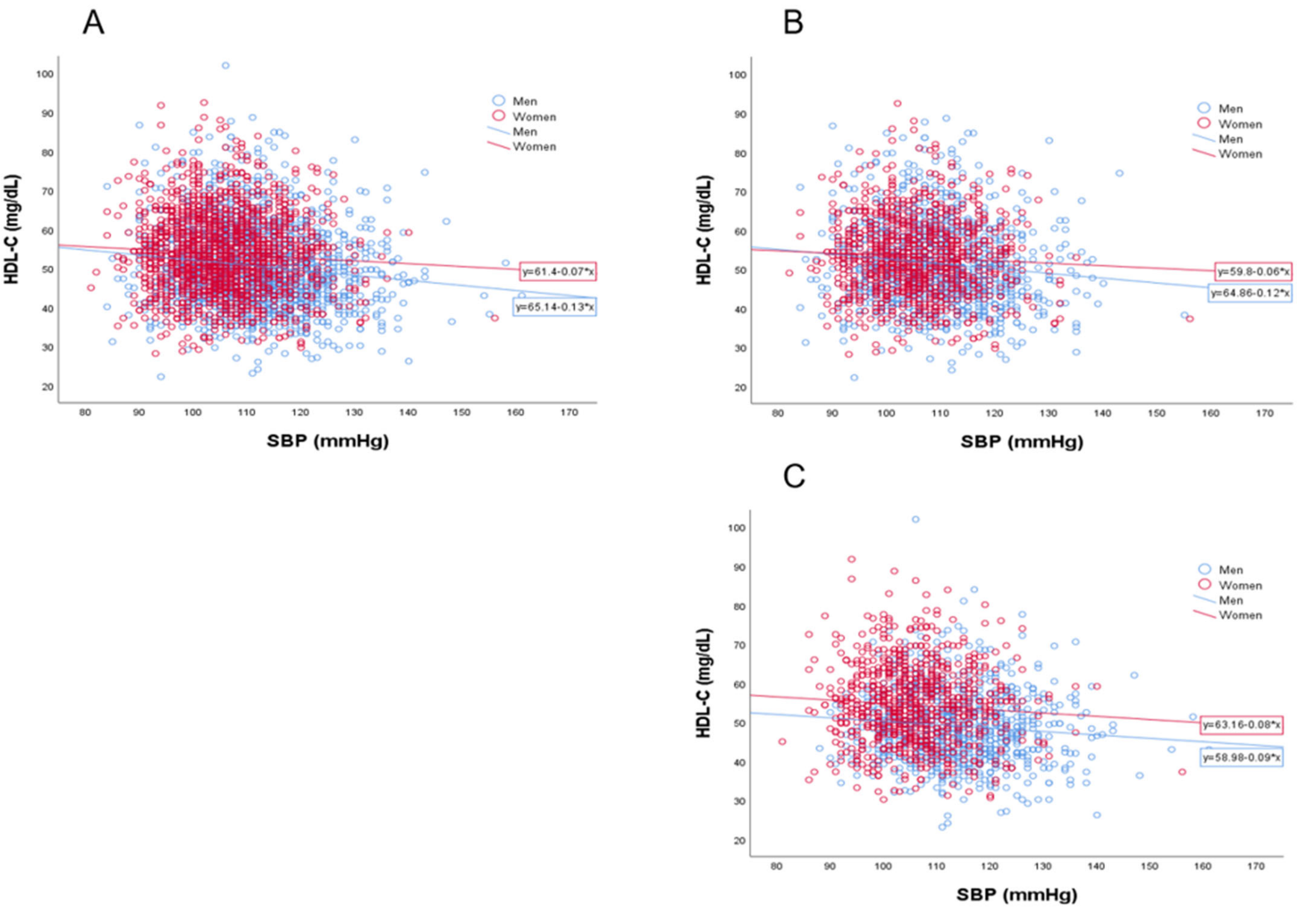

Figure S7. Pearson's correlation analysis of HDL-C and SBP in 10–19 years old (A) or 10–15 years old (B) or 15–19 years old (C). In panel A (10–19 years-old) the men group showed negative correlation (r = −0.133, $p < 0.001$) and the women group showed negative correlation (r = −0.065, $p = 0.009$). In panel B (10–15 years-old) the men group showed negative correlation (r = −0.115, $p = 0.0001$) and the women group showed no correlation (r = −0.058, $p = 0.073$). In panel C (15–19 years-old) the men group showed negative correlation (r = −0.100, $p = 0.003$) and the women group showed negative correlation (r = 0.074, $p = 0.034$).

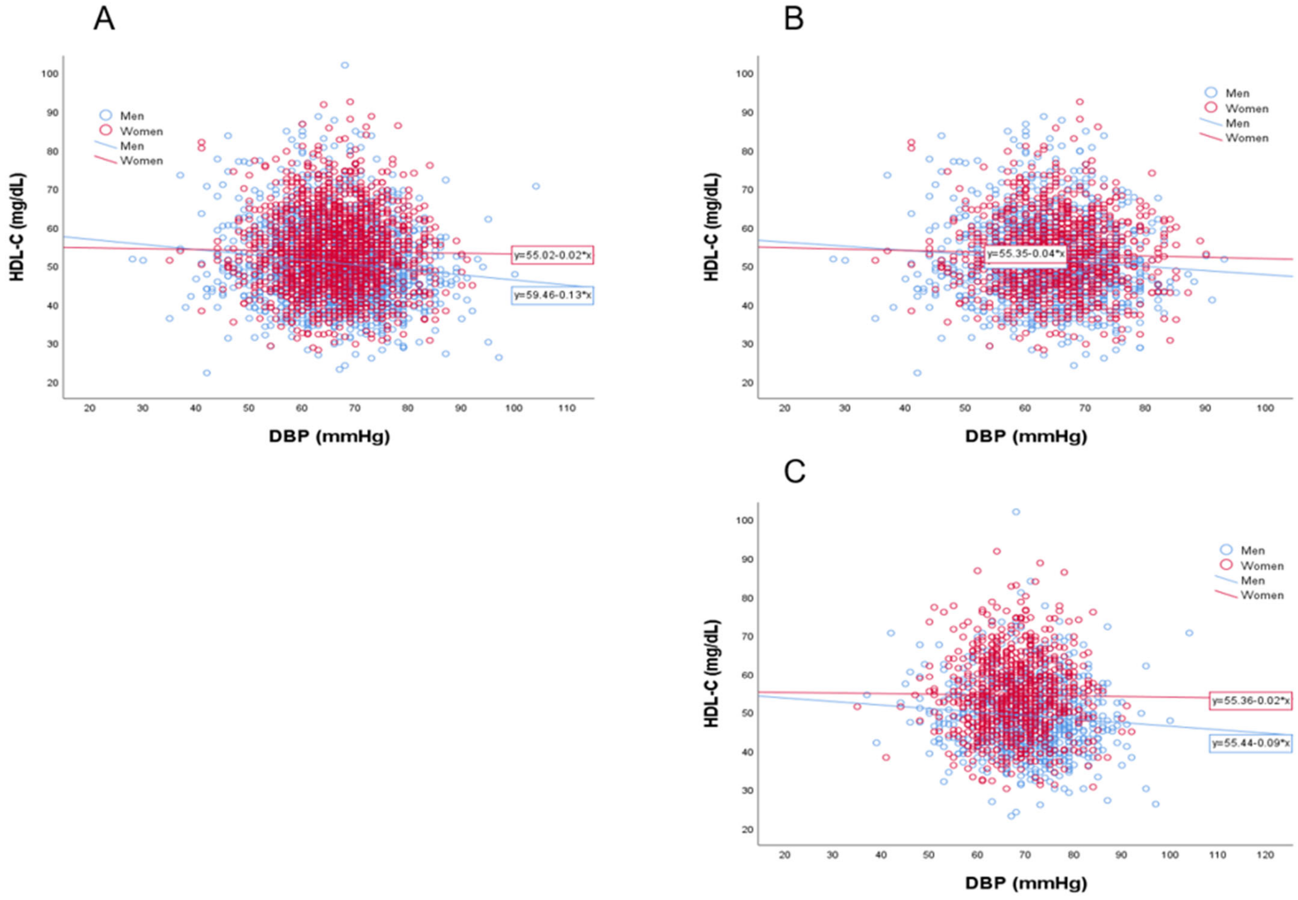

Figure S8. Pearson's correlation analysis of HDL-C and DBP in 10–19 years old (A) or 10–15 years old (B) or 15–19 years old (C). In panel A (10–19 years-old) the men group showed negative correlation (r = −0.122, $p < 0.001$) and the women group showed no correlation (r = −0.016, $p = 0.516$). In panel B (10–15 years-old) the men group showed negative correlation (r = −0.087, $p = 0.003$) and the women group showed no correlation (r = −0.029, $p = 0.365$). In panel C (15–19 years-old) the men group showed negative correlation (r = −0.088, $p = 0.009$) and the women group showed no correlation (r = −0.011, $p = 0.745$).
